# Fosfomycin Protects Mice From *Staphylococcus aureus* Pneumonia Caused by α-Hemolysin in Extracellular Vesicles by Inhibiting MAPK-Regulated NLRP3 Inflammasomes

**DOI:** 10.3389/fcimb.2019.00253

**Published:** 2019-07-15

**Authors:** Yanan An, Yang Wang, Jiuyu Zhan, Xudong Tang, Keshu Shen, Fengge Shen, Chao Wang, Wenjing Luan, Xuefei Wang, Xueyan Wang, Mingyuan Liu, Qingchuan Zheng, Lu Yu

**Affiliations:** ^1^Laboratory of Theoretical and Computational Chemistry, International Joint Research Laboratory Nano-Micro Architecture Chemistry, Key Laboratory for Zoonosis Research, Ministry of Education, Institute of Theoretical Chemistry, Institute of Zoonosis, College of Veterinary Medicine, Department of Infectious Diseases, First Hospital of Jilin University, Jilin University, Changchun, China; ^2^Key Lab for New Drugs Research of TCM in Shenzhen, Research Institute of Tsinghua University in Shenzhen, Shenzhen, China; ^3^Jilin Hepatobiliary Hospital, Changchun, China; ^4^Jiangsu Co-innovation Center for the Prevention and Control of Important Animal Infectious Diseases and Zoonoses, Yangzhou, China

**Keywords:** Fosfomycin, *Staphylococcus aureus*, Hla, SMVs, NLRP3 inflammasome

## Abstract

α-Hemolysin (Hla) is a significant virulence factor in *Staphylococcus aureus* (*S. aureus*)-caused infectious diseases such as pneumonia. Thus, to prevent the production of Hla when treating *S. aureus* infection, it is necessary to choose an antibiotic with good antibacterial activity and effect. In our study, we observed that Fosfomycin (FOM) at a sub-inhibitory concentration inhibited expression of Hla. Molecular dynamics demonstrated that FOM bound to the binding sites LYS 154 and ASP 108 of Hla, potentially inhibiting Hla. Furthermore, we verified that staphylococcal membrane-derived vesicles (SMVs) contain Hla and that FOM treatment significantly reduced the production of SMVs and Hla. Based on our pharmacological inhibition analysis, ERK and p38 activated NLRP3 inflammasomes. Moreover, FOM inhibited expression of MAPKs and NLRP3 inflammasome-related proteins in *S. aureus* as well as SMV-infected human macrophages (MΦ) and alveolar epithelial cells. *In vivo*, SMVs isolated from *S. aureus* DU1090 (an isogenic Hla deletion mutant) or the strain itself caused weaker inflammation than that of its parent strain 8325-4. FOM also significantly reduced the phosphorylation levels of ERK and P38 and expression of NLRP3 inflammasome-related proteins. In addition, FOM decreased MPO activity, pulmonary vascular permeability and edema formation in the lungs of mice with *S. aureus*-caused pneumonia. Taken together, these data indicate that FOM exerts protective effects against *S. aureus* infection *in vitro* and *in vivo* by inhibiting Hla in SMVs and blocking ERK/P38-mediated NLRP3 inflammasome activation by Hla.

## Introduction

*Staphylococcus aureus* (*S. aureus*) is an opportunistic and resilient a major bacterial pathogen in humans that colonizes mucosal surfaces (Nair et al., 2014; Lehar et al., 2015). It can cause a significant disease burden and can grow on nearly all tissues of the host, including the skin, nares, bones, joints, and muscles and even in the heart and lungs (Alonzo and Torres, 2014; Zhang et al., 2015). *S. aureus* is not only the cause of biofilm-related diseases, but it can also induce the occurrence of human sepsis and contaminate food (Otto, 2008; Cheung et al., 2014). Thus, *S. aureus* infection is a major global health and social burden.

Many factors contributing to the rise of *S. aureus* as a formidable pathogen involve cell surface proteins and exotoxins, which alter the host immune system in a variety of ways. The most important *S. aureus* toxin is α-hemolysin (Hla), a water-soluble monomeric protein that is a cytolytic exotoxin secreted by most pathogenic strains of *S. aureus* and has a molecular mass of 33.2 kDa (Tanaka et al., 2011). Hla binds to target membranes, such as those on monocytes and macrophages (Mϕ), forms membrane-inserted heptameric pores, and subsequently allows water, ions, and other small molecular weight molecules to enter and exit the cells. Previous studies have found that Hla plays an important role in the infection of most tissues, such as the bloodstream and pulmonary and intraperitoneal tissues (Bramley et al., 1989; Kebaier et al., 2012; Rauch et al., 2012; Foletti et al., 2013). Moreover, recent evidence indicates that *S. aureus*-secretes α-hemolysin as a staphylococcal membrane-derived vesicle (SMV)-associated form (Hong et al., 2014). SMVs are spherical complexes that are extracellular vesicular structures and include proteins and toxins, among other substances (Lee et al., 2009).

Mϕs play pivotal roles in the adaptive immune and innate immune responses and are crucial for the recognition and scavenging of microbial pathogens (Aderem and Ulevitch, 2000). Moreover, when Mϕs are infected, stimulators can activate mitogen-activated protein kinases (MAPKs) and can participate in different cellular activities, such as mitosis, cell differentiation and cell survival or apoptosis, among other processes (Pearson et al., 2001). MAPKs (JNK, ERK and p38) regulate immune responses and inflammation in Mϕs (Ajizian et al., 1999). Furthermore, the central role of the NLR family in the immune system has become increasingly appreciated in recent years; the most important is the NLRP3 inflammasome (Franchi et al., 2009), which plays an essential role in mediating caspase-1 activation and subsequent cleavage of pro-inflammatory mediators to promote phagocyte recruitment in host defense. NLRP3-mediated inflammasome activation occurs in response to diverse molecular entities, including bacteria (Melehani et al., 2015), viruses (Wang et al., 2014), fungi (Mao et al., 2014), components of dying cells (Mariathasan et al., 2004), crystal particles (Mulay et al., 2014), and DNA (Kailasan Vanaja et al., 2014), among other factors. Thus, the *S. aureus* virulence factor Hla might induce NLRP3-related signaling to stimulate the activation of caspase-1 and programmed necrosis (Craven et al., 2009).

Fosfomycin (FOM) is an antibacterial drug that is bactericidal *in vitro* and *in vivo*, especially against methicillin-resistant *S. aureus* (MRSA) strains (Poeppl et al., 2011). Furthermore, due to its non-toxic and pharmacological properties, FOM is considered to be a promising clinical medication (Grif et al., 2001). Therefore, further studies of its pharmacological mechanism are warranted.

In this study, we determined that a sub-inhibitory concentration FOM had an inhibitory effect on the secretion of Hla and protected against mouse pneumonia caused by *S. aureus* and its SMVs *in vivo*. Thus, we further assessed the possible anti-inflammatory molecular mechanism of FOM in Mϕ and a mouse model of *S. aureus* pneumonia.

## Materials and Methods

### Ethics Statement

The mice were housed in micro-isolator cages and were provided diet and water *ad libitum*. The laboratory temperature and relative humidity were maintained at 24 ± 1°C and 40–80%. All animal experiments were carried out according to the experimental practices and standards approved by the Animal Welfare and Research Ethics Committee at Jilin University (no: IZ-2009-008). The experimental protocols were reviewed and approved by the committee. All animals received humane care in compliance with the Guide for the Care and Use of Laboratory Animals published by the US National Institutes of Health (NIH Publication No. 85-23, revised 1996). To minimize animal suffering, all animal experiments were performed under isoflurane anesthesia.

### Antibodies and Chemicals

The primary antibodies used for western blotting (anti-ASC antibody, anti-Caspase-1 p20 antibody, anti-IL-18 antibody, anti-IL-1β antibody, anti-Pro-Caspase-1 antibody, anti-Pro-IL-18 antibody, anti-Pro-IL-1β antibody anti-NLRP3 antibody, anti-p-ERK antibody, anti-p-P38 antibody, anti-p-JNK antibody), in the study were all obtained from Cell Signaling Technology (1:1,000 Massachusetts, USA). The secondary antibodies used for western blotting were obtained from Beyotime (1:1,000 Jiangsu, China). Inhibitors were obtained from MCE (shanghai, China). Other chemical reagents were obtained from Dingguo Changsheng (Beijing, China).

### Strains and Growth Conditions

*S. aureus* 8325-4, DU1090, SA113, Xen29, and RN6390 were used in this study. Bacterial were grown in Tryptic Soy Broth (TSB) (Oxoid, Basingstoke, UK) at 37°C. FOM was obtained from Sigma-Aldrich.

### Preparation of Cells

The Mϕ THP-1 cells and alveolar epithelial cells (MLE-12) were obtained from the cell bank of the Chinese Academy of Sciences (Shanghai, China). Phagocytic cells were grown in the RPMI 1640 and with 10% FBS, 100 Uml-1 penicillin, and 100 μg ml^−1^ streptomycin at 37°C in the presence of 5% CO_2_. Ten nanogram per milliliter phorbol myristate acetate were used to induced THP-1 cells differentiated to Mϕ. MLE-12 cells were cultured in DMEM basal medium supplemented with 10% FBS, 100 U ml^−1^ penicillin and 100 μg ml^−1^ streptomycin at 37°C in the presence of 5% CO_2_.

### Antimicrobial Susceptibility Testing

To determine the minimum inhibitory concentrations (MICs) of FOM, according to CLSI guidelines, we performed microbroth dilution assays. The MIC was lowest inhibitory concentration, which showing no growth in sight. The minimum bactericidal concentration (MBC) was identified as the lowest concentration in the agar plate to show no microbial survival.

### Agar Plate Hemolysis Tests

Sheep blood agar plates were prepared using defibrinated sheep blood (Becton Dickinson, 5% in TSB, 15 ml per plate) and were used to analyze direct or synergistic hemolysis with bacterial cultures, purified Hla, and purified SMVs with or without FOM treatment. Plates were incubated for 24 h before analysis.

### Hemolysis Assay

*S. aureus* strains 8325-4/DU1090 were incubated in TSB at 37°C until to an optical density at 600 nm (OD_600_) = 2.5 and with or without the addition of FOM. Then centrifuged the cultures. The supernatant was collected. One hundred microliter including different concentration supernatant were pre-incubated in Eppendorf tubes with defibrinated rabbit erythrocytes (25 μL) and 875 μL hemolytic buffer (20 mM CaCl_2_, 0.125 M NaCl) at 37°C for 30 min. Hemolytic buffer served as a negative control. After centrifugation, we removed the supernatants, and measured the absorbance at 450 nm.

### Scanning Electron Microscopy

Scanning electron microscopy (SEM) was used to analyze the number of spherical vesicles associated with the *S. aureus* surface. 2.5% glutaraldehyde were used to fixed cells at 4°C for 30 min, then 1% osmium tetroxide were used to fixed, and graded ethanol series were used to dehydrated, critical-point dried, and then sputter coating a gold film to covered cells. The scanning electron microscope (Hitachi S-3400N, Japan) were used to analyze the specimens.

### Vesicle Extraction Separation

*S. aureus*-secreted membrane-derived vesicles were isolated using previously described methods (Prados-Rosales et al., 2014). Briefly, *S. aureus* 8325-4/DU1090 and FOM-pretreated *S. aureus* 8325-4/DU1090 were incubated at 37°C overnight in an orbital shaker at 200 rpm. Strains were grown in 200 ml of TSB medium to an optical density at 600 nm (OD_600_) = 2.5. After a series of gradient centrifugation and filtration, the remaining supernatant was ultracentrifuged in a Beckman low-temperature ultracentrifuge (Beckman, USA) at 120,000 g for 1 h at 4°C to obtain the membrane vesicles.

### Molecular Dynamics and Binding Mode of FOM With Hla

The Hla crystal structure (PDB code: 4IDJ) was used as the dissociative monomer of this protein. In this conformation, residues 130 to 140 were unresolved and modeled with MODELLER9.14 (Sali and Blundell, 1993). Then, a 15-ns molecular dynamics (MD) simulation was constructed with AMBER11 (Wang et al., 2006) software to optimize this monomer conformation. Subsequently, clustering analysis was used to obtain a representative structure. Chain A of the heptamer crystal structure (PDB code: 7AHL) was used to obtain the separated monomer. The molecular docking calculations were performed using the AUTODOCK (Morris et al., 2009) package. All of the non-polar hydrogen atoms were removed, while the polar ones were retained. The box sizes were 40 × 40 × 40 A^3^, and the grid spacing was 0.375 A, ensuring that all critical residues around the binding side were considered. FOM was docked into the box of each protein structure. The docking parameters were 100 CA runs, 15,000,000 maximum evaluations, and 270,000 generations. The conformation that had the lowest binding free energy was chosen as the representative structure.

### Cytokine Release

After incubation of THP-1 cells and MLE-12 cells with *S. aureus* 8325-4/DU1090 (E:T ratio, 5:1) or their vesicles (50 μg ml^−1^) (Gurung et al., 2011) for 6 h with or without 4 μg ml^−1^ FOM at 37°C, the samples were centrifuged to collected the supernatant and were stored at −20°C until testing for human interleukin 1β (IL-1β; eBioscience, San Diego, CA, USA) and human interleukin 18 (IL-18; R&D Systems, Minneapolis, MN, USA) concentrations using Quantikine enzyme-linked immunosorbent assays (eBioscience). The bronchoalveolar lavage (BAL) fluid of mice was centrifuged, and supernatants were obtained for mouse interleukin 1β (IL-1β; eBioscience, San Diego, CA, USA) and mouse interleukin 18 (IL-18; MBL, Nagoya, Japan) measurements. Three independent experiments were performed.

### Determination of Cytotoxicity (CCK-8)

Cell counting Kit-8 (CCK-8) from Promega (Madison, USA) were used to evaluate the cytotoxic effects. 1 × 10^4^ cells were seeded in 96 well plates and then incubated with strain 8325-4, DU1090, their SMVs, FOM, and Hla for 48 h. Then, 10 μL of CCK-8 solution per well was added and according to the manual incubated the cells for 2–4 h. FLUO star Optima microplate reader (BMG Labtechnologies, Jena, Germany) were used to measure the absorbance at 450 nm.

### Western Blot Analysis

*S. aureus* strains 8325-4/DU1090 were incubated in TSB at 37°C until to an optical density at 600 nm (OD_600_) = 2.5 with or without the addition of FOM. Centrifuged the cultures with 8,500 g for 30 min at 4°C, and then co-incubated culture supernatants (900 μL) with 100 μL TCA for 12 h at 4°C. Centrifuged to collect the supernatants proteins. And the extracted SMVs were frozen and thawed repeatedly until broken. The protein was tested Hla by western blot analysis.

THP-1 cells (1 × 10^6^) were treated with *S. aureus* 8325-4/DU1090 (E:T ratio, 5:1), their vesicles (50 μg mL^−1^) or pure Hla (100 μg/mL, Sigma Aldrich) for 6 h with or without 4 μg mL^−1^ FOM at 37°C. And MLE-12 cells were treated with *S. aureus* 8325-4/DU1090 (E:T ratio, 5:1) with or without 4 μg mL^−1^ FOM for 6 h at 37°C. The culture supernatants of the cells were collected and the proteins in the culture supernatants were precipitated using 10% trichloroacetic acid (Sigma) to test the expression of active forms of caspase-1 p20, IL-1β, and IL-18. RIPA lysis buffer (Sigma, Missouri, USA), which containing 1 mM PMSF (Sigma, Missouri, USA) incubated with the collected cells on ice for 10 min. And 12,000 g centrifugation to obtain the supernatant of lysates to test the other related protein. Then for quantify the concentration of protein, the BCA protein assay kit (Beyotime, Jiangsu, China) were used following the instructions. The protein lysates were separated by 12% SDS-PAGE and then transferred to PVDF membranes (Beyotime, Jiangsu, China). The 5% skim milk as blocking buffer to soak the membranes for 2 h, and then the primary antibodies were used to incubated at 4°C overnight, followed by secondary antibodies for 2 h at RT. The enhanced chemiluminescence detection kit (Beyotime, Jiangsu, China) were used to detect the corresponding bands. And then collected the image by a Cano Scan Li DE 100 scanner (Canon, Tokyo, Japan).

### Mouse *S. aureus* or Vesicle Pneumonia Model

The mouse pneumonia model was performed as described previously (Ragle et al., 2010). In short, BALB/c male mice aged 6 weeks were inoculated with resuspended *S. aureus* (2 × 10^8^ CFUs per 50 μL) and vesicles (25 μg/mouse, equivalent to 2 × 10^8^ vesicles) (Prados-Rosales et al., 2011) through the left nare (*n* = 8 mice each group). To investigate the effects of FOM treatment, mice were separately administered 40 mg/kg FOM or PBS 2 h after *S. aureus* infection and then at 12-h intervals or were directly injected with vesicles for 24 h. After 24 h, infected mice were euthanized with anesthesia. The lungs were weighed and homogenized for the calculation of bacterial burden. For histopathologic analysis, formalin-fixed lung tissues were subjected to hematoxylin and eosin staining, and lung tissue sections were then visualized using an Olympus BX53 fluorescence microscope (Olympus, Tokyo, Japan) with a 20× objective lens. Intratracheal instillation was performed to collect BAL fluid with 400 μL of pre-cooled PBS. The lavage fluid was centrifuged, and the supernatants were used for cytokine measurements. Cell pellets were resuspended and stained with Wright's-Giemsa for total and differential cell counts. The lungs were removed and were stored at −80°C for western blot analysis.

### MPO Assay

Lung tissues were weighted and homogenized in cold PBS. The supernatants were collected and the MPO activity in lung tissues was detected by the MPO assay kits (Jiancheng Bioengineering Institute, Nanjing, China) according to the manufacturer's instructions.

### Measurement of Lung Vascular Permeability and Lung Water Content

Lung vascular permeability was measured in each experiment. The Evans blue assay was used to test vascular permeability in lung tissue and was performed as described previously (Radu and Chernoff, 2013; Pati et al., 2018; Zhou et al., 2019). Briefly, the mice received an intravenous injection of 1% Evans blue dye solution (Sigma, St. Louis, MO, USA). After 1 h, the mice were sacrificed and perfused via the right ventricle with 4°C PBS for 10 min to introduce the dye intravascularly, and the lung tissues were collected. The lungs were placed in 1 ml of formamide (Avantor, Center Valley, PA, USA) at 60°C for 24 h to extract the Evans blue dye. The lung tissues were then centrifuged at 2,000 rpm for 10 min, and the supernatants were collected. The concentrations of Evans blue dye in the supernatant were measured at an absorbance of 620 nm. For lung water content, the left lung was harvested and weighed to determine the wet weight in each group. The lungs were then dried to a constant weight at 60°C for 48 h and were weighed to determine the dry weight. The lung water content was calculated as the ratio of wet weight to dry weight.

### Statistical Analysis

All statistical analyses in this study were performed using SPSS Statistics 19.0 (IBM, USA). The data are representative of triplicate experiments and are presented as mean values ± SDs. Significance was assessed using Student's *t*-test. Multiple intervention experiments were compared with one-way ANOVA followed by Tukey's *post-test* correction. *P*-values of 0.05 or less were considered statistically significant.

## Results

### FOM Inhibits the Hemolytic Activity of Hla *in vitro*

**Figure 1:**
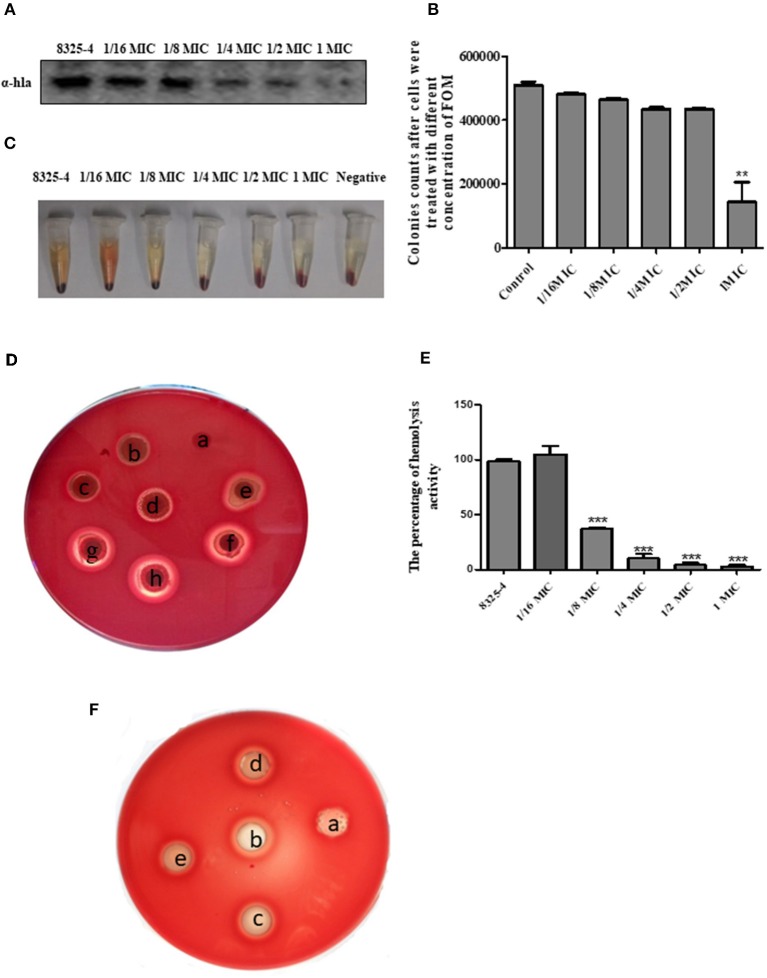
FOM inhibits the hemolytic activity of Hla *in vitro*. **(A)** *S. aureus* 8325-4 were incubated in TSB at 37°C to a post-exponential growth phase (OD _600nm_ = 2.5) with or without FOM. The supernatant protein was collected, proteins were tested by western blot analysis. **(B)** Colony counts of *S. aureus* 8325-4 treated with different concentrations of FOM. **(C)** Hemolytic ability of *S. aureus* 8325-4 supernatant assay. (a: TSB; b: 2 MIC; c: 1 MIC, d: 2^−1^ MIC; e: 4^−1^ MIC; f: 8^−1^ MIC; g: 16^−1^ MIC; h: Supernatant of *S. aureus* 8325-4). **(D)** Hemolytic ability of *S. aureus* 8325-4 supernatant assay with the red cells of rabbit co-incubation. **(E)** Supernatant of *S. aureus* 8325-4 hemolytic ability assay by measuring the absorbance at 450 nm. **(F)** Hemolytic ability of α-Hla and SMVs and with or without FOM assay. (a: TSB; b: α-Hla; c: α-Hla ± FOM, d: SMVs; e: SMVs ± FOM). Data are the means ± standard errors derived from three experiments. * *P* < 0.05, ** *p* < 0.01, *** *p* < 0.001, significantly different from *S. aureus* 8325-4.

The drug susceptibility assay demonstrated that FOM possesses antibacterial and bactericidal activities against 5 representative *S. aureus* strains (RN6390, Xen29, SA113, 8325-4, and DU1090) (Table 1); their MICs and MBCs were 16 and 32 ~ 256 μg ml^−1^. Moreover, western blot analysis showed that FOM inhibits expression of Hla of *S. aureus* 8325-4 at a sub-inhibitory concentration in a dose-dependent manner (Figures 1A,B). The results indicated that the reduced expression of Hla was caused by FOM, though the number of bacteria did not decrease notably. We also found that the hemolytic activity of *S. aureus* 8325-4 was attenuated by FOM in a dose-dependent manner (Figures 1C,D). Furthermore, we verified the supernatant hemolytic capacity by measurement of the absorbance at 450 nm with a spectrophotometer, and the results showed that FOM significantly reduced hemolytic effects at more than 1/8 MIC (Figure 1E). To verify whether FOM is able to inhibit the hemolytic activity induced by Hla and SMVs, we performed a blood plate experiment. The results showed that purified Hla and SMVs caused significant hemolysis and that FOM significantly inhibited hemolysis caused by purified Hla and purified SMVs (Figure 1F). These results demonstrated that FOM had a good anti-Hla effect.

**Table 1:** Activities of FOM against S. aureus, as determined by microbroth dilution assays and agar plate method.

**Strain**	**MIC**	**MBC**
RN6390	16	64
Xen29	16	256
SA113	16	64
8325-4	16	128
DU1090	16	64

### FOM Inhibited the Production of *S. aureus* SMVs and the Amount of Hla Mediated by SMVs

**Figure 2:**
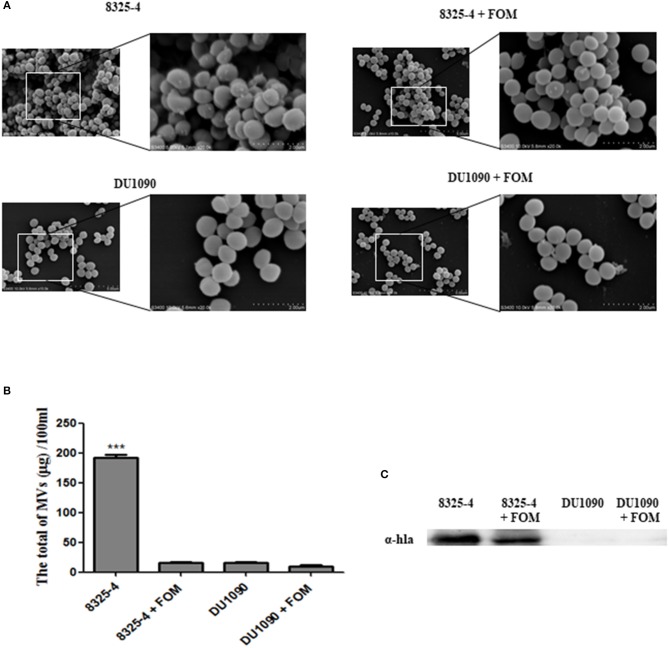
Release of SMVs by *S. aureus* with or without FOM treatment. **(A)** Scanning electron microscopy was used to observe the FOM-treated or untreated *S. aureus* SMVs production (10K×/20K×). **(B,C)** The SMVs were extracted and quantified, western blot was used to detect the Hla content. Data are the means ± standard errors derived from three experiments. * *P* < 0.05, ** *p* < 0.01, *** *p* < 0.0001, significantly different from *S. aureus* 8325-4.

Recent evidence indicated that *S. aureus* secretes extracellular vesicles containing Hla (Lee et al., 2009). Thay *et al*. also reported that Hla was tightly associated with the SMVs (Thay et al., 2013). In our study, we observed the production of SMVs on the surface of *S. aureus*, whereas treatment with FOM significantly inhibited MV production (Figure 2A). Moreover, we compared the amounts of the extracted SMVs from *S. aureus* and found that strain 8325-4 pretreated with FOM and strain DU1090 had significantly decreased production of vesicles (Figure 2B). In addition, we verified the existence of Hla in membranous proteins extracted from the isolated SMVs of strain 8325-4, and pretreatment with FOM significantly reduced the amount of Hla from SMVs; however, no Hla was found in strain DU1090, with or without FOM treatment (Figure 2C). These results implied that Hla was related to SMVs and that FOM treatment inhibited the production of SMVs and the amount of Hla mediated by SMVs.

### FOM Inhibited Separated Monomer of Hla

**Figure 3:**
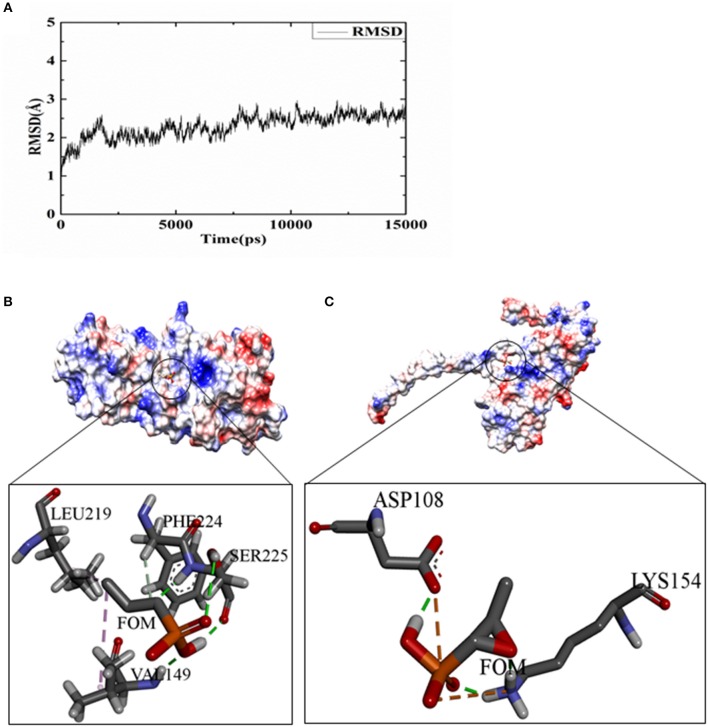
FOM binding assay for Hla and docking studies. **(A)** The RMSD curve of the backbone atoms referenced to the first frame. **(B)** The details of the binding sides of the FOM-dissociative monomer. **(C)** The details of the binding sides of the FOM-separated monomer. The green dotted lines represent hydrogen bond interactions; pink represent hydrophobic interaction; and orange represent salt bridge interaction.

To analyze the possible inhibitory mechanisms of FOM on Hla, we explored the direct binding of FOM to Hla by a ligand-binding assay using docking. A 15-ns molecular dynamics (MD) simulation was carried out on the dissociative monomer of the protein, whose gap residues were modeled with the MODELLER software package. The root-mean-square deviation (RMSD) from the starting structure is an important criterion for the convergence of the system. As shown in Figure 3A, the RMSD of this system remained stable during the entire simulation time, especially the last 5 ns. The 15 ns of the trajectory were divided into five clusters (named C0, C1, C2, C3, and C4) by the average-linkage algorithm (Prager and Wilson, 1978). In clustering analysis, one snapshot was chosen as the representative structure in each cluster (Table 2). C4 was the most robust cluster in the system and was thus chosen as the typical structure for the subsequent molecular docking.

**Table 2:** Populations of every clusters of each complex.

**Clusters**	**C0(%)**	**C1(%)**	**C2(%)**	**C3(%)**	**C4(%)**
Percentages	5.5	7.7	24.8	13.1	48.9

To obtain some energetic and structural insight into the inhibitory mechanisms of this inhibitor, molecular dockings were further performed using AUTODOCK software. The three-dimensional structures of the complexes of FOM-dissociative monomer and FOM-separated monomer showed that FOM bound to the binding sites of PHE 224 and LEU 219, mediated by hydrophobic interaction, and to the binding sites of SER225 and VAL 149, mediated by hydrogen bond interactions in the dissociative monomer of Hla (Figure 3B), and the binding sites of LYS 154 and ASP 108 were mediated by hydrogen bond interactions and salt bridge interactions in the separated monomer of Hla (Figure 3C). The docking simulation was successful, with significant scores. In the complex of FOM-dissociative monomer, the binding energy predicted by Molecular Mechanics–Generalized Born Surface Area (MM-GBSA) calculation was −1.56 kcal/mol, while the value in the complex of FOM-separated monomer was −2.85 kcal/mol. The latter had a lower binding energy than did the former, which may be the reason for the anti-virulence effect of FOM against *S. aureus* Hla via the direct inhibition of the separated monomer not formation of heptamer.

### FOM Inhibits the MAPK Pathway in *S. aureus*- and SMV-Infected Cells

**Figure 4:**
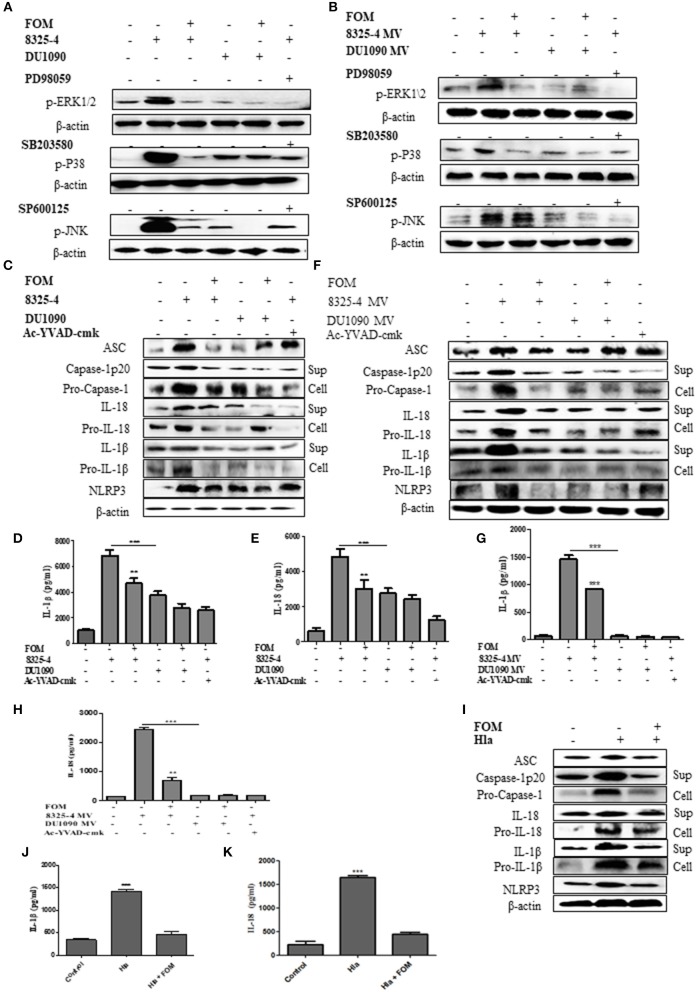
FOM inhibits MAPK and NLRP3 inflammasome protein activation in *S. aureus* or SMVs-infected Mϕ. **(A)** Western blot was used to test phosphorylation of JNK, ERK1/2, and p38 in THP-1 cells with ERK inhibitor (PD98059), p38 inhibitor (SB203580), and JNK inhibitor (SP600125). **(B)** SMVs were produced by *S. aureus* 8325-4/DU 1090 or by *S. aureus* 8325-4/DU 1090 pretreated with FOM to treat THP-1 cells. Western blot was used to analyze the p-JNK, p-ERK1/2, and p-P38 proteins expression. **(C)** Western blot was used to analyze the activation of NLRP3 inflammasomes-related protein (ASC, caspase-1p 20, Pro-caspase-1, IL-1β, Pro-IL-1β, IL-18, Pro-IL-18, and NLRP3) in THP-1 cells in the both culture supernatant or cell lysate. **(D,E)** Quantitative detection of IL-18 and IL-1β secretion by ELISA. **(F)** The activation of NLRP3 inflammasomes in the both culture supernatant and cell lysate in THP-1 cells after treatment with SMVs. **(G,H)** Quantitative detection of IL-18 and IL-1β secretion by ELISA. **(I)** The activation of NLRP3 inflammasomes in the both culture supernatant and cell lysate in Mϕ after treatment with Hla. **(J,K)** Quantitative detection of IL-18 and IL-1β secretion by ELISA. Sup is culture supernatant and cell are cell lysate. Data are means ± standard errors derived from three experiments. * *P* < 0.05, ** *p* < 0.01, *** *p* < 0.0001.

MAPKs have been reported to play an important role in inflammation (Yu et al., 2013; Ghonime et al., 2014; Okada et al., 2014). To investigate the effect of FOM on the MAPK-mediated inflammation by *S. aureus*- or SMV-stimulated THP-1 cells, we investigated the changes in the phosphorylation levels of JNK, ERK1/2, and p38 in THP-1 cells. The expression levels of p-ERK1/2, p-P38, and p-JNK in THP-1 cells were increased by *S. aureus* 8325-4/DU1090 (Figure 4A and Figures S2A–C) or the SMVs produced by strain 8325-4 (Figure 4B and Figures S2D–F). Moreover, strain DU1090 had a weaker stimulation than strain 8325-4; however, the SMVs produced by strain DU1090 barely induced the expression of MAPK proteins (Figures 4A,B and Figures S2A–F). FOM inhibited the expression of MAPK proteins in Mϕ induced by *S. aureus* 8325-4/DU1090 (Figure 4A and Figures S2A–C), with the same effect observed on FOM-pretreated strain 8325-4 SMVs (Figure 4B and Figures S2D–F). To verify the effect of FOM against MAPK-related inflammation induced by *S. aureus* or its SMVs in Mϕ, specific inhibitors were used. The results showed that FOM could significantly inhibit the phosphorylation of JNK, ERK1/2, and p38 and achieve the same effect as all of the inhibitors in strain 8325-4- or SMV-infected Mϕ, respectively (Figures 4A,B). The results showed that FOM is able to inhibit the MAPK-mediated inflammation in Mϕ induced by *S. aureus* or its SMVs.

To investigate the effect of FOM on the MAPK-mediated inflammation in *S. aureus*-stimulated MLE-12 cells, western blots were used to evaluate changes in the phosphorylation levels of JNK, ERK1/2, and p38, and the expression levels of p-ERK1/2, p-P38, and p-JNK increased after stimulation by *S. aureus* 8325-4/DU1090 (Figures S5A,H). However, the effect of strain DU1090 was much weaker than that of strain 8325-4. The results showed that FOM inhibited the MAPK pathway in MLE-12 cells induced by *S. aureus* 8325-4/DU1090 (Figures S5A,H).

### FOM Inhibits NLRP3 Inflammasome Activation in *S. aureus*- and SMV-Infected Cells

As mentioned above, NLRP3 inflammasomes produced by phagocytes are crucial for defending against *S. aureus* (Craven et al., 2009). In this study, we investigated the changes in NLRP3 inflammasome related-proteins (ASC, caspase-1 p20, Pro-caspase-1, IL-1β, Pro-IL-1β, Pro-IL-18, IL-18, and NLRP3) in Mϕ in culture supernatant or cell lysate after treatment with *S. aureus* or SMVs with or without FOM. The results showed that *S. aureus* 8325-4/DU1090 or SMVs induced expression of NLRP3 inflammasome-related proteins in THP-1 cells (Figures 4C–F and Figures S3A–H). The levels of those NLRP3 inflammasome-related proteins in Mϕ induced by strain DU1090 were lower than those of strain 8325-4; however, the SMVs produced by strain DU1090 barely induced inflammasome-related protein expression. Furthermore, the secretion levels of IL-1β and IL-18 induced by *S. aureus* 8325-4/DU1090 were lower after FOM treatment (Figures 4D,E). Similarly, the levels of IL-1β and IL-18 in cells treated with SMVs from strain 8325-4 were significantly increased compared with cells treated with SMVs produced by FOM pretreatment (Figures 4G,H). Ac-YVAD-cmk, a caspase-1 inhibitor, inhibited the levels of caspase-1 p20, Pro-caspase-1, IL-1β, Pro-IL-1β, IL-18, and Pro-IL-18 in strain 8325-4- or SMV-infected Mϕ, respectively (Figures 4D–F and Figures S3I–P). Previous reports observed that NLRP3 inflammasomes in human monocytic cells could be activated by *S. aureus* Hla, along with the activation of caspase-1 (Munoz-Planillo et al., 2009). In our study, the levels of NLRP3 inflammasome-related proteins in Mϕ induced only by *S. aureus* Hla were inhibited by FOM (Figures 4I–K and Figures S3Q–X). These results suggested that FOM could inhibit NLRP3 inflammasomes induced by *S. aureus*, SMVs or Hla *in vitro*.

We then investigated the effect of FOM on the NLRP3 inflammasome-related proteins in *S. aureus*-stimulated MLE-12 cells. The results showed that *S. aureus* 8325-4/DU1090 induced the expression of caspase-1 p20, Pro-caspase-1, IL-1β, Pro-IL-1β, ASC, IL-18, Pro-IL-18, and NLRP3 (Figures S5B,I). The levels of these NLRP3 inflammasome related-proteins in MLE-12 cells induced by strain DU1090 were lower than those of strain 8325-4. FOM inhibited expression of NLRP3 inflammasome-related proteins in MLE-12 cells induced by *S. aureus* 8325-4/DU1090 (Figures S5B,I), and the secretion levels of IL-1β and IL-18 induced by *S. aureus* 8325-4/DU1090 were lower after FOM treatment (Figures S5C,D).

### MAPKs Mediate Expression of NLRP3 Inflammasome Proteins in Cells

**Figure 5:**
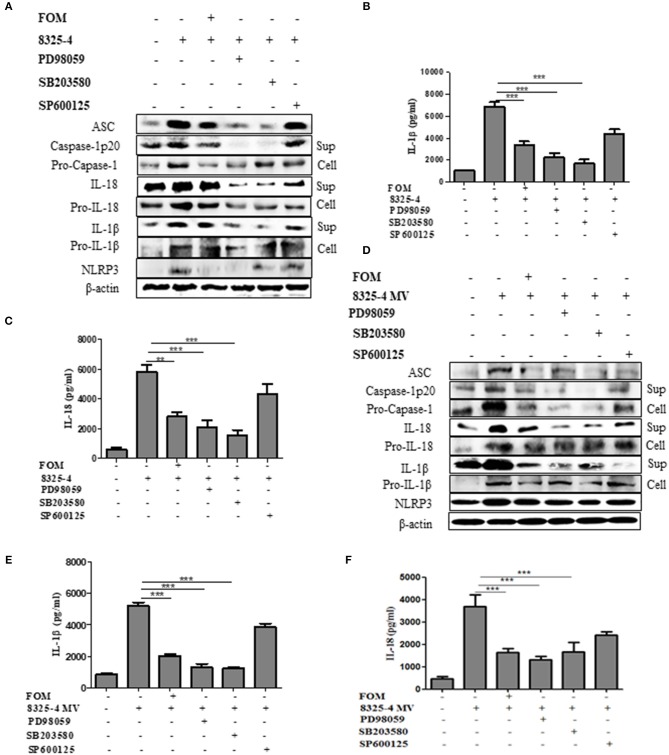
MAPKs mediated the activation of NLRP3 inflammasome in Mϕ. **(A)** Western blot was used to test the activation of NLRP3 inflammasomes-related proteins (ASC, caspase-1 p20, Pro-caspase-1, IL-1β, Pro-IL-1β, IL-18, Pro-IL-18 and NLRP3) in Mϕ with ERK inhibitor (PD98059), p38 inhibitor (SB203580), and JNK inhibitor (SP600125) in the both culture supernatant or cell lysate. **(B,C)** Quantitative detection of IL-18 and IL-1β secretion by ELISA. **(D)** The activation of NLRP3 inflammasomes (ASC, caspase-1 p20, Pro-caspase-1, IL-1β, Pro-IL-1β, IL-18, Pro-IL-18, and NLRP3) in the both culture supernatant and cell lysate in Mϕ were analysis by western blot and three inhibitors were used. **(E,F)** Quantitative detection of IL-18 and IL-1β secretion by ELISA. Sup is culture supernatant and cell are cell lysate. Data are means ± standard errors derived from three experiments. * *P* < 0.05, ** *p* < 0.01, *** *p* < 0.0001.

A further experiment was performed to examine the relationship between MAPKs and NLRP3 inflammasomes. We used specific inhibitors of P38 (SB203580), ERK (PD98059), and JNK (SP600125) to pretreat cells, among which SB203580 and PD98059, but not SP600125, significantly inhibited the production of ASC, caspase-1 p20, Pro-caspase-1, IL-1β, Pro-IL-1β, IL-18, Pro-IL-18, and NLRP3 induced by *S. aureus* (Figures 5A–C and Figures S4A–H, S5E–G,J) or SMVs (Figures 5D–F and Figures S4I–P). These results showed that FOM inhibited the p-P38- and p-ERK1/2-mediated NLRP3 inflammasomes activated by *S. aureus* or SMV infection in Mϕ and MLE-12 cells, but not JNK-mediated inflammasomes.

To demonstrate that the expression or inhibition of the above proteins was induced by strain 8325-4, DU1090, or their SMVs, pure Hla and FOM rather than their cytotoxic effects on the cells, we evaluated the optimal doses of strain 8325-4, DU1090, their SMVs, and pure Hla for cytotoxicity in the cells. The results showed that the optimal doses were not cytotoxic to the cells' survival (Figure S1).

To verify whether FOM directly blocks inflammation, we treated THP-1 cells with peptidoglycan and found that peptidoglycan could significantly induce the expression of MAPKs and NLRP3 inflammasome-related proteins, while FOM did not significantly inhibit the expression of these proteins (Figures S6A,C). Furthermore, to demonstrate that FOM could directly inhibit Hla-mediated activation of inflammasomes, we treated cells with LPS for 4 h to prime them and then added Hla; after 4 h, we then used FOM to treat the cells, culture supernatants and cell lysates were harvested, and western blot were used to detect the expression levels of NLRP3 inflammasome-related proteins. The results showed that FOM significantly inhibited the expression of inflammasome-related proteins induced by Hla (Figures S6B,D). In summary, FOM inhibits NLRP3 inflammasomes by inhibiting Hla.

### FOM Protects Mice Against *S. aureus* Pneumonia

**Figure 6:**
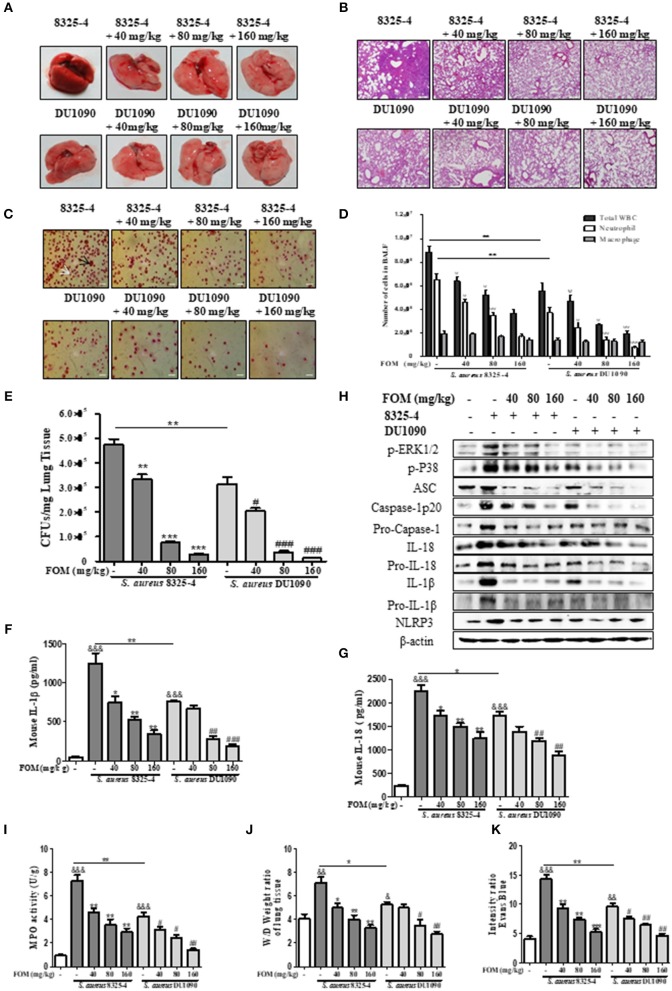
FOM protects mouse *S. aureus* pneumonia *in vivo*. **(A,B)** Gross pathological changes **(A)** and histopathology **(B)** of *S. aureus*-infected lung tissue with or without FOM treatment. Tissues were stained with HE (10×). **(C)** Wright's-Giemsa stained smear of peritoneal fluid from infected mice with FOM treatment. Neutrophil (white arrow) and Mϕ (black arrow) are presented (20×). **(D)** The total of WBC, neutrophil and Mϕ in the bronchoalveolar lavage (BAL) fluid of infected mice with FOM treatment were counted stained with Wright's-Giemsa. **(E)** Bacterial burden in the lungs of infected mice with or without FOM treatment ^ψ^ *p* < 0.05, ^ψψ^ *p* < 0.01, ^ψ*ψψ*^ *p* < 0.001 compared with 8325-4 treated group. **(F,G)** The level of IL-1β and IL-18 in the bronchoalveolar lavage (BAL) fluid of infected mice with FOM treatment was detected by Elisa. **(H)** The levels of p-ERK, p-P38 and NLRP3 inflammasome protein in *S. aureus* 8325-4/DU1090-infected lungs with FOM treatment. **(I)** Effects of FOM on MPO activity of *S. aureus* -induced lung inflammation in mice. **(J)** Lung water content was calculated as the ratio of wet weight to dry weight, **(K)** vascular leakage in lung tissue was measured via injecting Evans blue dye. ^&^ *p* < 0.05, ^&&^ *p* < 0.01, ^&*&&*^ *p* < 0.001 compared with Control group, * *p* < 0.05, ** *p* < 0.01, *** *p* < 0.001 compared with 8325-4 treated group, ^#^ *p* < 0.05, ^*##*^ *p* < 0.01, ^*###*^ *p* < 0.001 compared with DU1090 treated group. Data are means ± standard errors derived from three experiments.

Due to the important role of FOM *in vitro*, we continued to study its protective effects *in vivo*. Gross pathological images of *S. aureus*-infected lung tissue indicated that the lung tissue of *S. aureus* 8325-4/DU1090-infected mice experienced a color change from dark pink to light pink with FOM treatment in a dose-dependent manner (Figure 6A). Histopathology images revealed that strain DU1090 caused weaker inflammation than that of its parent strain, 8325-4 (Figures 6A,B), and the lung tissue showed a significant aggregation of inflammatory cells (dark blue or purple) in the alveolar space of the *S. aureus* 8325-4-infected group (Figure 6B). However, FOM treatment significantly reduced the pulmonary inflammation, such as accumulation of inflammatory cells in the alveolar space (Figure 6B). Neutrophils and Mϕ were distinguished in Wright's-Giemsa-stained BAL fluid from infected mice with FOM treatment (Figure 6C). The total numbers of WBC, neutrophils and Mϕ in the BAL fluid of infected mice were counted with Wright's-Giemsa stain (Figure 6D). As Figure 6D shows, the total numbers of WBCs and neutrophils were significantly decreased by FOM, while Mϕs were not decreased by FOM, in the BAL fluid of *S. aureus* 8325-4/DU1090-infected mice. Moreover, the total numbers of WBCs and neutrophils in strain DU1090-infected mice were significantly decreased compared with those of strain 8325-4-infected mice (Figure 6D). The colony counts of *S. aureus*-infected lungs results indicated that FOM treatment significantly reduced CFUs in the lungs of *S. aureus* 8325-4/DU1090-infected mice in a dose-dependent manner (Figure 6E). The CFUs in the lungs of strain DU1090-infected mice were significantly lower than those of strain 8325-4-infected mice after infection, with or without FOM treatment.

Furthermore, FOM significantly decreased the levels of IL-1β and IL-18 in the BAL fluid of *S. aureus* 8325-4/DU1090-infected mice, while 40 mg/kg FOM only significantly decreased the level of IL-18 in the BAL fluid of strain DU1090-infected mice (Figures 6F,G). At the same time, we investigated the levels of p-PEK1/2- and p-38-mediated NLRP3 inflammasomes in the lung in response to FOM during *S. aureus* infection. The results showed that FOM significantly decreased the levels of p-PEK1/2, p-P38, ASC, caspase-1 p20, Pro-caspase-1, IL-1β, Pro-IL-1β, IL-18, Pro-IL-18, and NLRP3 in the lungs of *S. aureus* 8325-4/DU-1090-infected mice (Figure 6H and Figures S7A–J). All of these results indicated that FOM had a good protective effect against mouse pneumonia caused by *S. aureus* Hla *in vivo*.

### SMVs Induce Pneumonia in Mice

**Figure 7:**
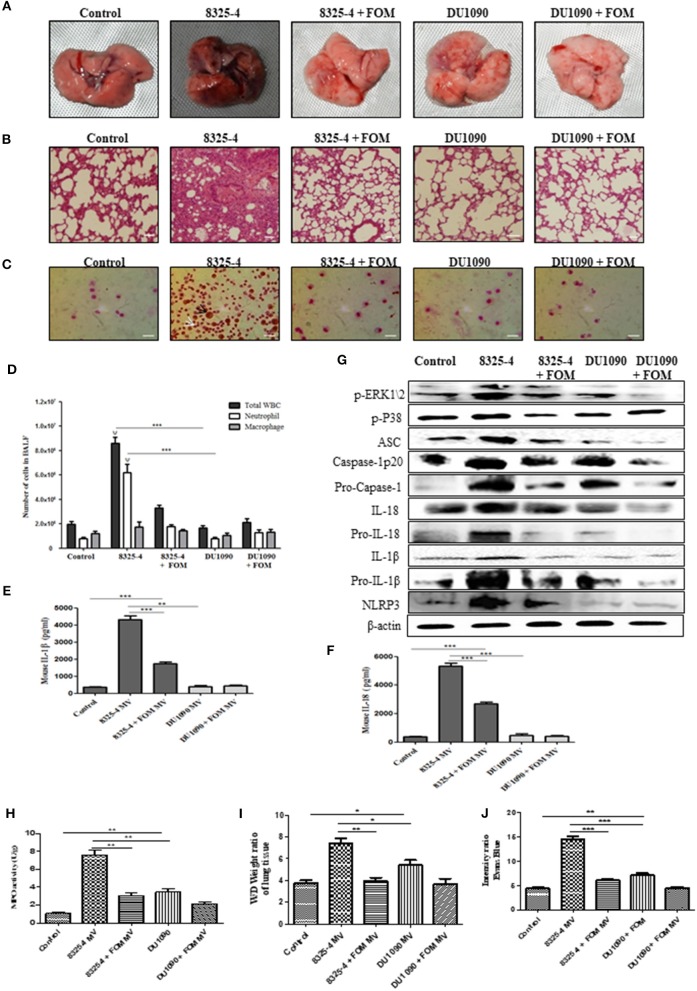
SMVs induced the occurrence of pneumonia in mice *in vivo*. **(A,B)** Gross pathological changes **(A)** and histopathology **(B)** of pretreated with or without FOM. Tissues were stained with HE (10×). **(C)** Wright's-Giemsa stained smear of peritoneal fluid from infected mice. Neutrophil (white arrow) and Mϕ (black arrow) are presented (20×). **(D)** The total of WBC, neutrophil and Mϕ in the bronchoalveolar lavage (BAL) fluid of infected mice with FOM treatment were counted stained with Wright's-Giemsa. ^ψ^ *p* < 0.05, ^ψψ^ *p* < 0.01, ^ψ*ψψ*^ *p* < 0.001 compared with FOM treated group. **(E,F)** The level of IL-1β and IL-18 in the bronchoalveolar lavage (BAL) fluid of infected mice was detected by Elisa. **(G)** The levels of p-ERK, p-P38, and NLRP3 inflammasome protein in different SMVs-infected lungs. **(H)** Effects of FOM on MPO activity of SMVs-induced lung inflammation in mice. **(I)** Lung water content was calculated as the ratio of wet weight to dry weight, **(J)** vascular leakage in lung tissue was measured via injecting Evans blue dye. * *p* < 0.05, ** *p* < 0.01, *** *p* < 0.001 compared with the Control group. Data are means ± standard errors derived from three experiments.

To further study the pathogenesis of Hla, we extracted the SMVs of *S. aureus* and used them to induce pneumonia in mice. The pathology images from lung tissues infected with SMVs of strain 8325-4 showed a much darker pink color compared with those of lungs infected with the SMVs of strain DU1090 or the control group. However, FOM pretreatment could rescue the darker pink lungs caused by SMVs from strain 8325-4 (Figure 7A). Histopathological sections revealed that the SMVs produced by strain 8325-4 caused a significant accumulation of inflammatory cells (dark blue or purple) in the alveolar space of the lung tissue compared with strain DU1090 (Figure 7B). However, FOM pretreatment of strain 8325-4 SMVs and strain DU1090 SMVs did not result in a marked accumulation of cellular infiltrates in the alveolar space (Figure 7B). To explore the distribution of neutrophils and Mϕ, we examined Wright's-Giemsa-stained BAL fluid from infected mice with SMV treatment (Figure 7C). The results showed that compared with Mϕ, the WBCs and neutrophils were significantly increased in strain 8325-4 SMV-infected mice. Moreover, the total numbers of WBCs and neutrophils in strain DU1090 SMVs or FOM-pretreated *S. aureus* 8325-4/DU 1090 SMVs-infected mice were significantly decreased compared with strain 8325-4 SMV-infected mice. Moreover, there were no significant differences in the total numbers of WBCs and neutrophils between the control and strain DU1090 SMV-treated mice (Figure 7D); all of these results were the same as those in *S. aureus*-infected mice. Further, we examined the expression levels of inflammatory factors, such as IL-1β and IL-18, in the BAL fluid of SMV-infected mice. The results showed that strain DU1090 SMVs significantly decreased the levels of IL-1β and IL-18 compared with strain 8325-4 SMVs. In addition, the levels of IL-1β and IL-18 in FOM-pretreated *S. aureus* 8325-4/DU1090 SMVs were markedly decreased compared with the SMV-treated groups (Figures 7E,F). Previous reports have indicated that the MAPK family was a potential target for anti-inflammatory therapeutics (Park et al., 2012; Okada et al., 2014); therefore, we tested the relationships between them. The results showed that the levels of p-PEK1/2, p-P38, ASC, caspase-1 p20, Pro-caspase-1, IL-1β, Pro-IL-1β, IL-18, Pro-IL-18, and NLRP3 in the lungs of strain 8325-4 SMV-infected mice were significantly increased compared with the pretreated FOM groups, and there was no significant difference in the levels of protein expression between the control and strain DU1090 SMVs (Figure 7G and Figures S7K–T). In summary, all of these results reflected the same phenomenon as caused by *S. aureus* infection. These results indicate that SMVs are a direct form of *S. aureus* Hla expression and release its toxicity, while FOM has a significant inhibitory effect on Hla production.

### FOM Attenuates *S. aureus*- and SMV-Induced MPO Activity, Vascular Permeability and Alveolar Edema in the Lungs of Mice With Pneumonia

The effects of FOM on lung MPO activity induced by *S. aureus* or SMVs were detected in the present study. As shown in Figures 6I, 7H, *S. aureus* or SMV treatment increased the lung MPO activity compared with the normal control group. In contrast, the levels of lung MPO activity decreased significantly after treatment with FOM (Figures 6I, 7H). We then investigated the effects of FOM on vascular permeability and alveolar edema. Lung water content, expressed as wet/dry weight, was significantly increased in *S. aureus*- or SMV-infected mice, and it decreased significantly after FOM treatment (Figures 6J, 7I). Evans blue dye was used to determine the changes in alveolar permeability, and we found that FOM decreased Evan's Blue dye extravasation compared with *S. aureus*- or SMV-infected mice (Figures 6K, 7J).

## Discussion

*S. aureus* infection is characterized by a high complication rate and a high probability of recurrence of infection, and some strains are resistant to antibiotic treatment. Previous reports have shown that sub-inhibitory concentrations of some antibiotics can influence the pathogenesis of infection by affecting the expression of virulence factors of *S. aureus* (Bernardo et al., 2004; Stevens et al., 2007). As mentioned above, Hla is a necessary virulence factor for *S. aureus* toxicity. Our results showed that FOM inhibited the hemolytic activity and bacterial protein expression level of Hla in a dose-dependent manner at a concentration lower than the MIC; at the concentration equal to the MIC, FOM also completely inhibited the hemolytic activity and the bacterial protein expression level of Hla (Figure 1). These results may indicate that FOM can kill bacteria and inhibit the expression of the bacterial virulence protein Hla.

Previous research has proven that *S. aureus* SMVs can function as carriers to deliver functional Hla to other cells (Thay et al., 2013). Immunogold-EM results revealed that gold particles showed significant deposition where vesicle structures were destroyed (Thay et al., 2013). Those results were consistent with previous studies on *Campylobacter jejuni* lethal distending toxin (CDT), *Escherichia coli* α-hemolysin (HlyA), and hemolysin of enterohemorrhagic *E. coli* (EHEC) isolates (EHEC-Hly) (Balsalobre et al., 2006; Aldick et al., 2009; Lindmark et al., 2009). Our results implied that the increased production of SMVs was associated with the release of toxins and that FOM inhibited *S. aureus* Hla production, mediated by the inhibition of SMVs (Figure 2).

Until now, some compounds have inhibited the hemolytic activity of Hla, but without anti-*S. aureus* activity (Ragle et al., 2010). We think that these compounds were not the most optimal clinical application drugs. The point of using antibiotics is to inhibit the expression of virulence factors and to have good antibacterial activity for treating severe infections, such as those due to Hla-producing *S. aureus*; these antibiotics, such as FOM, not only kill the bacteria but also inhibit the toxin-induced pathogenicity. If the Hla inhibitor had no antibacterial activity, the invading bacteria would recover and continue to produce and release new toxin as soon as the inhibitor administration was stopped. In addition, our molecular dynamics simulations further confirmed that FOM inhibited the activity of Hla by binding to the binding sites of LYS 154 and ASP 108, mediated by hydrogen bond interactions and salt bridge interactions in the separated monomer of Hla (Figure 3). These findings might be the reason for the anti-virulence effect of FOM against *S. aureus* Hla, that is, by directly inhibiting Hla heptamer formation.

MAPKs (ERK1/2, JNK, and P38) are members of the serine/threonine protein kinases family, and mediate basic biological processes and participate in the responses of cells to external stress signals (Yu et al., 2013; Ghonime et al., 2014). Increased activity of MAPKs, in particular p38, makes them potential targets for anti-inflammatory therapeutics (Okada et al., 2014). Inhibitors against the JNK and p38 pathways have been reported, and clinical data indicate that the inhibitors have significant anti-inflammatory activity (Park et al., 2012). Our results showed that FOM treatment significantly reduced the levels of phosphorylated ERK1/2, JNK and P38 proteins in *S. aureus*-infected Mϕ (Figures 4A,B), indicating the FOM had anti-inflammatory effects by inhibiting the MAPK pathway.

To further research the anti-inflammatory effect of FOM in *S. aureus* infection, we evaluated the levels of NLRP3 inflammasomes in human Mϕ *in vitro*. NLRP3 is a member of the nucleotide-binding oligomerization domain-like receptor (NLR) family of pattern recognition receptors and plays an important role in response to bacterial and endogenous stimuli in the activation of caspase-1 and IL-1β secretion (Franchi et al., 2009). Our studies showed that FOM treatment significantly diminished the levels of the NLRP3 inflammasome-related proteins ASC, caspase-1, IL-1β, IL-18 and NLRP3 in *S. aureus* 8325-4/DU1090/their SMVs/Hla-treated Mϕ (Figures 4C–K). These findings were similar to a previous report that showed significant inhibition of IL-1β by FOM in LPS-stimulated THP-1 cells *in vitro* (Morikawa et al., 1996). These results showed that FOM could reduce NLRP3 inflammasome-mediated inflammation caused by *S. aureus* Hla.

In addition, to examine the relationship between MAPKs and NLRP3 inflammasomes induced by Hla, specific inhibitors were used to demonstrate that the MAPKs ERK and p38, but not JNK, regulated Hla-activated NLRP3 in Mϕ (Figure 5). Previous reports showed that inhibition of the ERK pathway and the activation of caspase-1 mediated by ATP or nigericin were markedly diminished in wild-type BMDMs (Park et al., 2015). Moreover, P38 senses a diversity of bacterial toxins, including proaerolysin (Huffman et al., 2004) and streptolysin O (Ratner et al., 2006) and toxin, and plays an important role in host defense. *S. aureus* α-toxin has been demonstrated to activate p38 (Husmann et al., 2006). Activation of JNK and p38 mediates a pro-inflammatory response and upregulates IL-1β transcription (Jandhyala et al., 2012). In our study, we showed that blockade of ERK1/2 or P38, but not JNK1/2, inhibited *S. aureus*-induced expression of NLRP3 proteins (Figures 5A–C).

Hla, an *S. aureus* pore-forming toxin, has been recognized as an essential virulence determinant for *S. aureus* pneumonia (Rauch et al., 2012). Hla-deficient mutant *S. aureus* strains displayed reduced virulence in mouse models of pneumonia (Bubeck Wardenburg et al., 2007). Recent research demonstrated that the activation of NLRP3 played an essential role in Hla-mediated *S. aureus* pneumonia in mice (Kebaier et al., 2012). Our study also verified that FOM alleviated pulmonary damage, as visualized by in gross pathological and histopathology images, markedly decreased WBC and neutrophil numbers, and significantly reduced levels of ASC, caspase-1, IL-18, IL-1β, NLRP3 induced by strain 8325-4, or SMVs in infected lungs (Figures 6, 7). These results showed that FOM had a good anti-inflammatory effect in mouse pneumonia caused by *S. aureus* Hla *in vivo*. The hemolytic activity of Hla plays an important role in the activation of host NLRP3 (Craven et al., 2009). This was in agreement with our result that FOM blocked MAPK-mediated NLRP3 inflammasome activation via inhibiting Hla. Previous reports indicated that Hla was able to activate inflammatory signals through inflammasome expression. To further verify this theory, Hla-deficient *S. aureus* was applied, but it failed to induce a significant infection (Bubeck Wardenburg et al., 2007). Previous studies indicated that passive immunization of mice with a monoclonal antibody protective against *S. aureus* pneumonia could inhibit NLRP3-mediated signaling *in vitro* (Ragle et al., 2010).

Indeed *S. aureus* α-, β-, and γ-Hla have been confirmed to be essential for the activation of the NLRP3 inflammasome (Craven et al., 2009; Munoz-Planillo et al., 2009; Kebaier et al., 2012). Kebaier et al. suggested that the α- and γ-toxins of *S. aureus* can perforate and penetrate the cell membrane and, thus, may be involved in the activation of the inflammasome (Kebaier et al., 2012). Moreover, knockout Hla strains significantly reduced the degree of pathogenicity in a variety of models, especially mouse *S. aureus* pneumonia, in a manner that was different from other toxins. Related research showed that IL-1β secretion could be induced by purified cell wall peptidoglycan (PGN) and antibiotic-killed *S. aureus* in human cells (Wang et al., 2000). Shimada et al. (2010) previously demonstrated that the activation of NLRP3 inflammasomes and the secretion of IL-1β can be stimulated by *S. aureus* PGN, which is a particulate and can be internalized via phagocytosis. All of these factors may explain why strain DU1090 also induced the weaker NLRP3 inflammasome activation (Figure 4C) found in this study. Moreover, FOM, a phosphonic acid antibiotic, plays an important role in the formation the bacterial cell wall (Woodruff et al., 1977). In this study, we found that FOM inhibited expression of NLRP3 inflammasomes (Figure 4C), which may be because PGN biosynthesis in strain DU1090 was also impeded by FOM.

In conclusion, FOM repressed expression of *S. aureus* Hla through a binding interaction and led to subsequent inhibition of ERK/P38-NLRP3 inflammasome activation, protecting mice against *S. aureus* pneumonia. Thus, the utility of FOM in inhibiting the expression of virulence factors and its antibacterial activity in treating severe *S. aureus* pneumonia are vitally important considerations for its clinical application.

## Ethics Statement

The mice were lived in micro-isolator cages and there free diet and water. The laboratory temperature and relative humidity were maintained at 24 ± 1°C and 40–80%. All animal experiments were carried out according to the experimental practices and standards approved by the Animal Welfare and Research Ethics Committee at Jilin University (no: IZ-2009-008). The experiments protocols were reviewed and approved by the committee. In order to minimize animal suffering, all animal experiments were performed under isoflurane anesthesia.

## Author Contributions

YA, YW, and JZ contributed to data acquisition, data analysis, data interpretation, and revised the manuscript. XT, KS, FS, CW, and WL contributed to data acquisition and data analysis. XFW, XYW, and ML contributed to data interpretation. QZ and LY contributed to data acquisition, data analysis, data interpretation, the writing and revision of the manuscript, and served as the principal investigators.

### Conflict of Interest Statement

The authors declare that the research was conducted in the absence of any commercial or financial relationships that could be construed as a potential conflict of interest.

## Supplementary Material

Figure S1 Cytotoxicity of the drug and strain used *in vitro*. Cytotoxicity of strains 8325-4, DU1090, their SMVs, FOM, and Hla concentrations used on the THP-1 cells.

Figure S2 FOM inhibits MAPKs pathway in *S. aureus*-or SMVs-infected cells. **(A,D)** The ratio of p-ERK/β-actin was calculated. **(B,E)** The ratio of p-P38/β-actin was calculated. **(C,F)** The ratio of p-JNK/β-actin was calculated. ^*^ *P* < 0.05, ^**^ *P* < 0.01, ^***^ *P* < 0.001 compared with the control groups in the same cell line. The data are representative of three experiments with similar results.

Figure S3 FOM inhibits NLRP3 inflammasome activation in *S. aureus* or SMVs -infected cells. **(A,I,Q)** The ratio of ASC/β-actin was calculated. **(B,J,R)** The ratio of Caspase-1 p20/β-actin was calculated. **(C,K,S)** The ratio of Pro-Caspase-1/β-actin was calculated. **(D,L,T)** The ratio of IL-18/β-actin was calculated. **(E,M,U)** The ratio of Pro-IL-18/β-actin was calculated. **(F,N,V)** The ratio of IL-1β/β-actin was calculated. **(G,O,W)** The ratio of Pro-IL-1β/β-actin was calculated. **(H,P,X)** The ratio of NLRP3/β-actin was calculated. ^*^ *P* < 0.05, ^**^ *P* < 0.01, ^***^ *P* < 0.001 compared with the control groups in the same cell line. The data are representative of three experiments with similar results.

Figure S4 MAPKs mediated the activation of NLRP3 inflammasome in cells. **(A,I)** The ratio of ASC/β-actin was calculated. **(B,J)** The ratio of Caspase-1 p20/β-actin was calculated. **(C,K)** The ratio of Pro-Caspase-1 /β-actin was calculated. **(D,L)** The ratio of IL-18/β-actin was calculated. **(E,M)** The ratio of Pro-IL-18/β-actin was calculated. **(F,N)** The ratio of IL-1β/β-actin was calculated. **(G,O)** The ratio of Pro-IL-1β/β-actin was calculated. **(H,P)** The ratio of NLRP3/β-actin was calculated. ^*^*P* < 0.05, ^**^*P* < 0.01, ^***^*P* < 0.001 compared with the control groups in the same cell line. The data are representative of three experiments with similar results.

Figure S5 FOM inhibits MAPK and NLRP3 inflammasome protein activation in *S. aureus* infected MLE-12. **(A)** Western blot was used to test phosphorylation of JNK, ERK1/2, and p38 in MLE-12 cells with ERK inhibitor (PD98059), p38 inhibitor (SB203580) and JNK inhibitor (SP600125). **(B)** The activation of NLRP3 inflammasomes-related protein (ASC, caspase-1 p20, Pro-caspase-1, IL-1β, Pro-IL-1β, IL-18, Pro-IL-18, and NLRP3) in MEL-12 cells. **(C,D)** Quantitative detection of IL-18 and IL-1β secretion by ELISA. **(E)** An ERK inhibitor (PD98059), p38 inhibitor (SB203580), and JNK inhibitor (SP600125) were used. Western blot was used to analyze the NLRP3 inflammasomes-related protein expression. **(F,G)** Quantitative detection of IL-18 and IL-1β secretion by ELISA. **(H)** The ratio of p-ERK/β-actin, p-P38/β-actin and p-JNK/β-actin were calculated. **(I,J)** The ratio of NLRP3 inflammasomes-related protein (ASC, caspase-1 p20, Pro-caspase-1, IL-1β, Pro-IL-1β, IL-18, Pro-IL-18, and NLRP3)/β-actin were calculated. Sup is culture supernatant and cell is cell lysate. Data are means ± standard errors derived from three experiments. ^*^ *P* < 0.05, ^**^ *p* < 0.01, ^***^ *p* < 0.0001.

Figure S6 FOM inhibits NLRP3 inflammasome by inhibiting Hla. **(A)** THP-1 cells were treated with peptidoglycan, and western blot were used to test the expression of MAPKs and NLRP3 inflammasome related proteins in the both culture supernatant and cell lysate. Sup is culture supernatant and cell are cell lysate. **(B)** THP-1 cells were first treated with LPS for 4 h, and then Hla was added, 4 h later FOM was added. Then the cells and culture supernatant were harvested and western blot were used to detect the expression of NLRP3 inflammasome related-protein. Sup is culture supernatant and cell are cell lysate. **(C)** The ratio of p-ERK/β-actin, p-P38/β-actin and p-JNK/β-actin and NLRP3 inflammasomes-related protein (ASC, caspase-1 p20, Pro-caspase-1, IL-1β, Pro-IL-1β, IL-18, Pro-IL-18, and NLRP3)/β-actin were calculated. **(D)** The ratio of NLRP3 inflammasomes-related protein (ASC, caspase-1 p20, Pro-caspase-1, IL-1β, Pro-IL-1β, IL-18, Pro-IL-18, and NLRP3)/β-actin were calculated. Data are means ± standard errors derived from three experiments. ^*^ *P* < 0.05, ^**^ *p* < 0.01, ^***^ *p* < 0.0001.

Figure S7 FOM protects mouse *S. aureus* pneumonia *in vivo*. **(A,K)** The ratio of p-ERK/β-actin was calculated. **(B,L)** The ratio of p-P38/β-actin was calculated. **(C,M)** The ratio of ASC/β-actin was calculated. **(D,N)** The ratio of Caspase-1 p20/β-actin was calculated. **(E,O)** The ratio of Pro-Caspase-1/β-actin was calculated. **(F,P)** The ratio of IL-18/β-actin was calculated. **(G,Q)** The ratio of Pro-IL-18/β-actin was calculated. **(H,R)** The ratio of IL-1β/β-actin was calculated. **(I,S)** The ratio of Pro-IL-1β/β-actin was calculated. **(J,T)** The ratio of NLRP3/β-actin was calculated. ^&^ *p* < 0.05, ^&&^ *p* < 0.01, ^&*&&*^ *p* < 0.001 compared with Control group, ^*^ *p* < 0.05, ^**^ *p* < 0.01, ^***^ *p* < 0.001 compared with 8325-4 treated group, ^#^ *p* < 0.05, ^*##*^ *p* < 0.01, ^*###*^ *p* < 0.001 compared with DU1090 treated group for *S. aureus* pneumonia *in vivo*. And ^*^ *P* < 0.05, ^**^ *P* < 0.01, ^***^ *P* < 0.001 compared with the control groups in the same cell line for SMVs pneumonia *in vivo*. The data are representative of three experiments with similar results.

## Abbreviations

Hla: α-Hemolysin
FOM: Fosfomycin
SMVs: *S. aureus* membrane-derived vesicles
NLR: NOD-like receptors
Mϕ: macrophage
*S. aureus*: *Staphylococcus aureus*
TLRs: Toll-like receptors
MRSA: methicillin-resistant *S. aureus*
PEP: phosphoenolpyruvate
TSB: Tryptic Soy Broth
MICs: minimum inhibitory concentrations
MBC: minimum bactericidal concentration
SEM: Scanning electron microscopy
PMSF: phenylmethyl-sulfonyl-fluoride
MD: Molecular dynamics
MAPKs: mitogen-activated protein kinases
CCK-8: Cell counting Kit-8
RMSD: root-mean-square deviation
MM-GBSA: Molecular Mechanics–Generalized Born Surface Area
CDT: cethal distending toxin
EHEC: enter hemorrhagic *E. coli*
EHEC-Hly: EHEC hemolysin
PGN: peptidoglycan
CLSI: Clinical and Laboratory Standards Institute
PVDF: polyvinylidene fluoride
BAL fluid: Bronchoalveolar Lavage fluid
WBCs: white blood cell
CFU: Colony-Forming Units.
