# Supplementary figures and images for: Fosfomycin Protects Mice From Staphylococcus aureus Pneumonia Caused by α-Hemolysin in Extracellular Vesicles by Inhibiting MAPK-Regulated NLRP3 Inflammasomes
Source: Front Cell Infect Microbiol. 2019 Jul 15;9:253. doi: 10.3389/fcimb.2019.00253 (PMC6644418; doi:10.3389/fcimb.2019.00253)

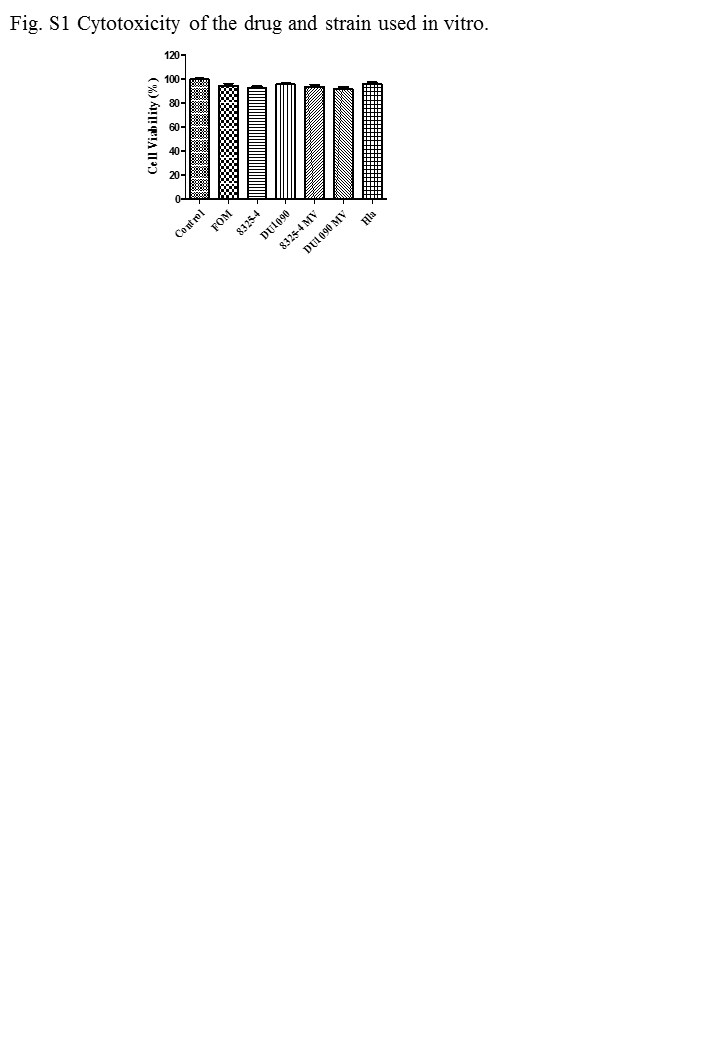

Supplement: Figure S1 — Cytotoxicity of the drug and strain used in vitro. Cytotoxicity of strains 8325-4, DU1090, their SMVs, FOM, and Hla concentrations used on the THP-1 cells. [file Image_1.tif]

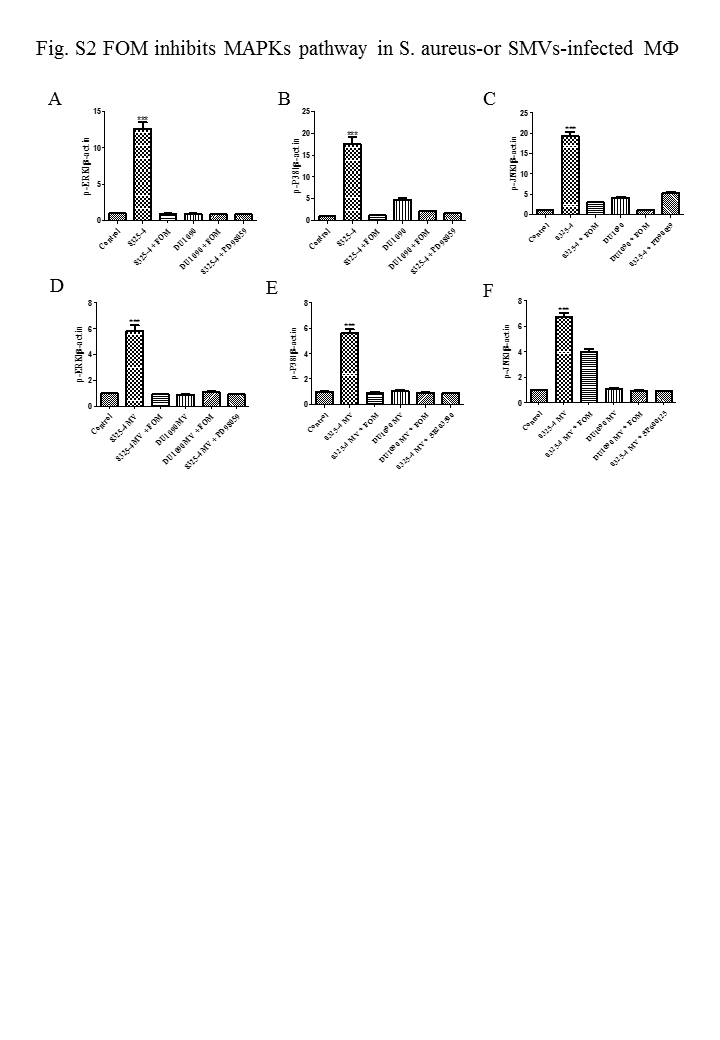

Supplement: Figure S2 — FOM inhibits MAPKs pathway in S. aureus-or SMVs-infected cells. (A,D) The ratio of p-ERK/β-actin was calculated. (B,E) The ratio of p-P38/β-actin was calculated. (C,F) The ratio of p-JNK/β-actin was calculated. * P < 0.05, ** P < 0.01, *** P < 0.001 compared with the control groups in the same cell line. The data are representative of three experiments with similar results. [file Image_2.tif]

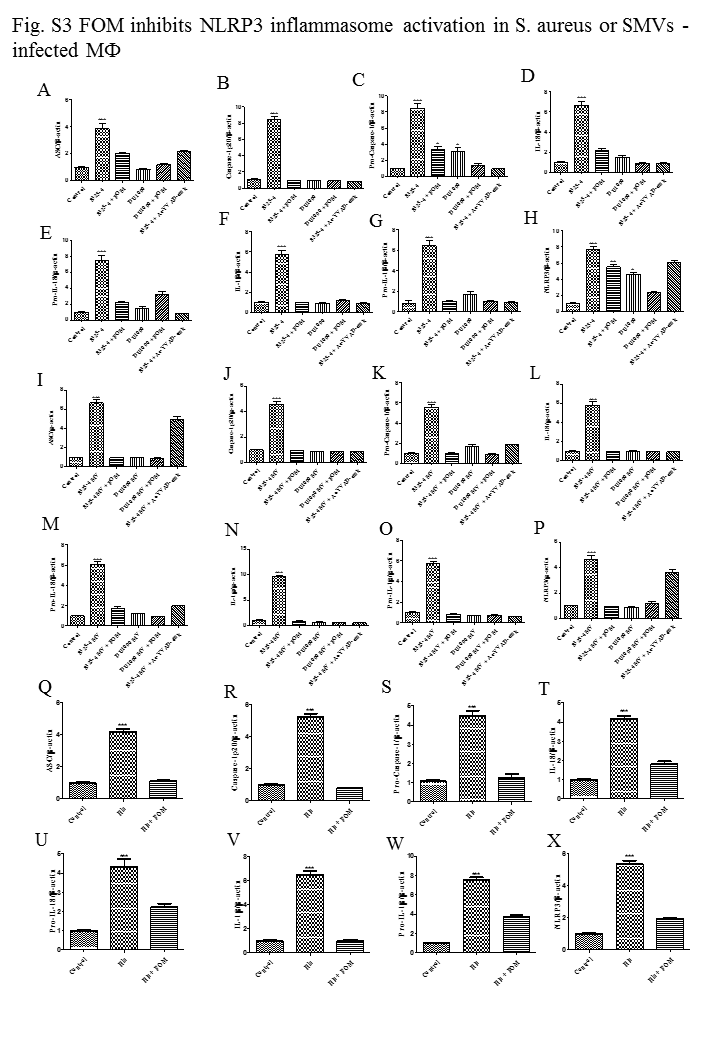

Supplement: Figure S3 — FOM inhibits NLRP3 inflammasome activation in S. aureus or SMVs -infected cells. (A,I,Q) The ratio of ASC/β-actin was calculated. (B,J,R) The ratio of Caspase-1 p20/β-actin was calculated. (C,K,S) The ratio of Pro-Caspase-1/β-actin was calculated. (D,L,T) The ratio of IL-18/β-actin was calculated. (E,M,U) The ratio of Pro-IL-18/β-actin was calculated. (F,N,V) The ratio of IL-1β/β-actin was calculated. (G,O,W) The ratio of Pro-IL-1β/β-actin was calculated. (H,P,X) The ratio of NLRP3/β-actin was calculated. * P < 0.05, ** P < 0.01, *** P < 0.001 compared with the control groups in the same cell line. The data are representative of three experiments with similar results. [file Image_3.tif]

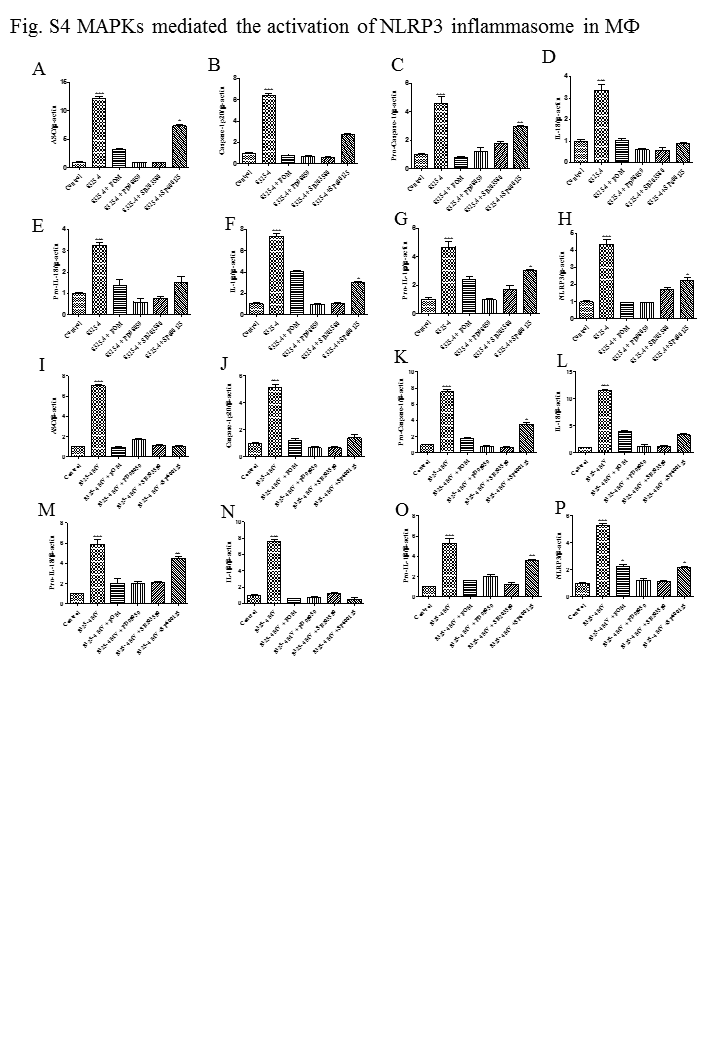

Supplement: Figure S4 — MAPKs mediated the activation of NLRP3 inflammasome in cells. (A,I) The ratio of ASC/β-actin was calculated. (B,J) The ratio of Caspase-1 p20/β-actin was calculated. (C,K) The ratio of Pro-Caspase-1 /β-actin was calculated. (D,L) The ratio of IL-18/β-actin was calculated. (E,M) The ratio of Pro-IL-18/β-actin was calculated. (F,N) The ratio of IL-1β/β-actin was calculated. (G,O) The ratio of Pro-IL-1β/β-actin was calculated. (H,P) The ratio of NLRP3/β-actin was calculated. *P < 0.05, **P < 0.01, ***P < 0.001 compared with the control groups in the same cell line. The data are representative of three experiments with similar results. [file Image_4.tif]

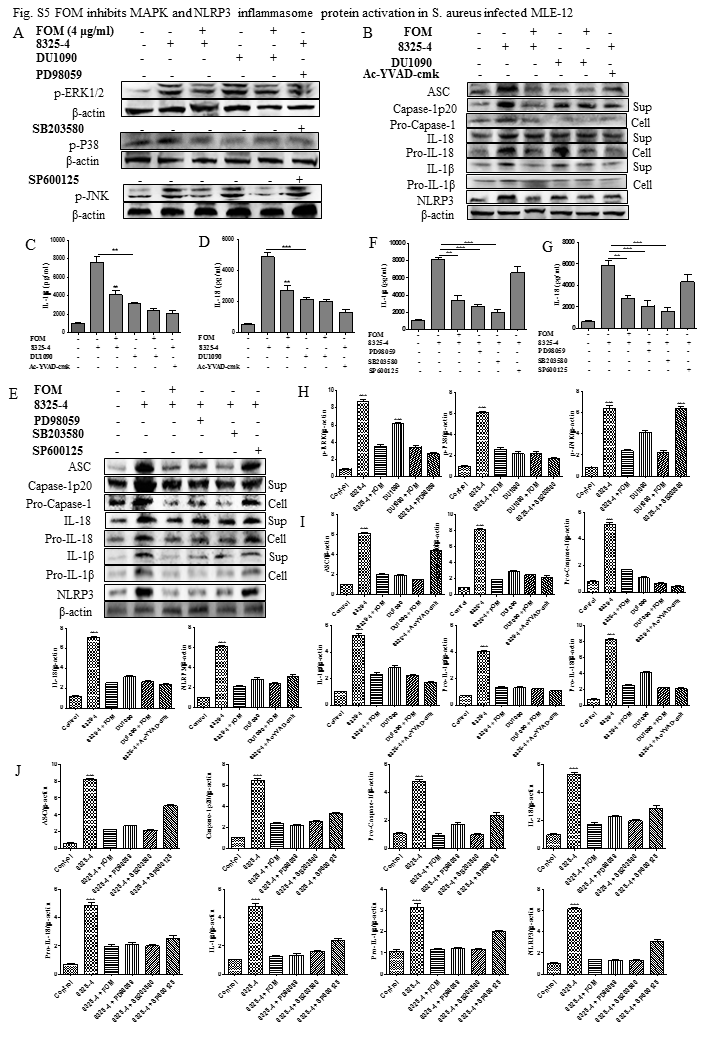

Supplement: Figure S5 — FOM inhibits MAPK and NLRP3 inflammasome protein activation in S. aureus infected MLE-12. (A) Western blot was used to test phosphorylation of JNK, ERK1/2, and p38 in MLE-12 cells with ERK inhibitor (PD98059), p38 inhibitor (SB203580) and JNK inhibitor (SP600125). (B) The activation of NLRP3 inflammasomes-related protein (ASC, caspase-1 p20, Pro-caspase-1, IL-1β, Pro-IL-1β, IL-18, Pro-IL-18, and NLRP3) in MEL-12 cells. (C,D) Quantitative detection of IL-18 and IL-1β secretion by ELISA. (E) An ERK inhibitor (PD98059), p38 inhibitor (SB203580), and JNK inhibitor (SP600125) were used. Western blot was used to analyze the NLRP3 inflammasomes-related protein expression. (F,G) Quantitative detection of IL-18 and IL-1β secretion by ELISA. (H) The ratio of p-ERK/β-actin, p-P38/β-actin and p-JNK/β-actin were calculated. (I,J) The ratio of NLRP3 inflammasomes-related protein (ASC, caspase-1 p20, Pro-caspase-1, IL-1β, Pro-IL-1β, IL-18, Pro-IL-18, and NLRP3)/β-actin were calculated. Sup is culture supernatant and cell is cell lysate. Data are means ± standard errors derived from three experiments. * P < 0.05, ** p < 0.01, *** p < 0.0001. [file Image_5.tif]

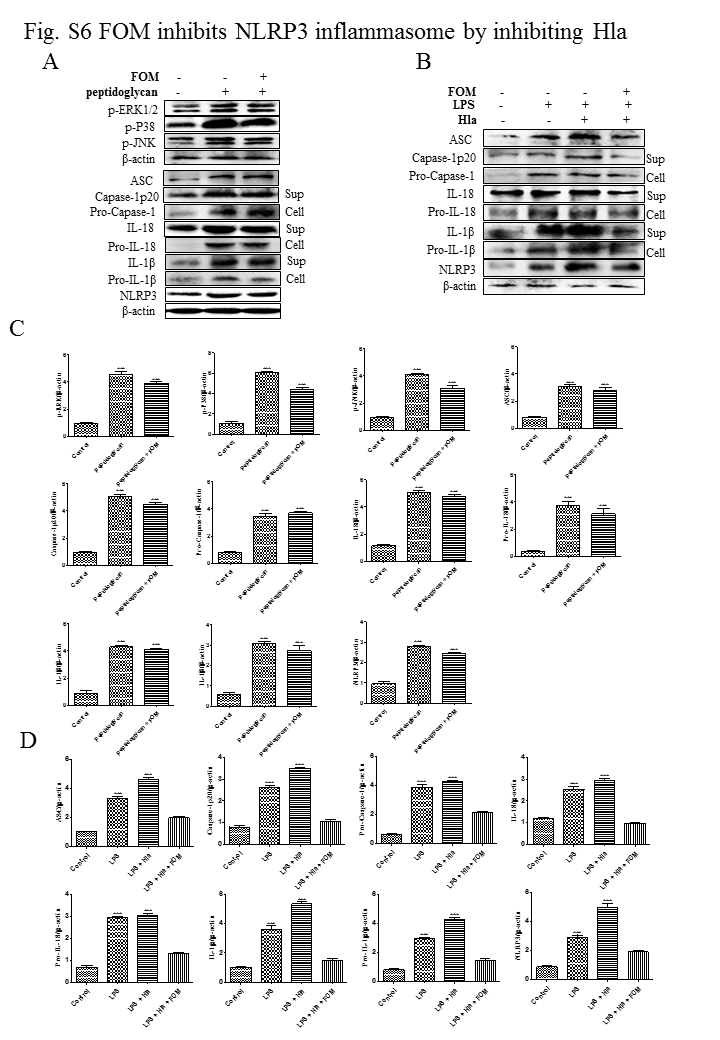

Supplement: Figure S6 — FOM inhibits NLRP3 inflammasome by inhibiting Hla. (A) THP-1 cells were treated with peptidoglycan, and western blot were used to test the expression of MAPKs and NLRP3 inflammasome related proteins in the both culture supernatant and cell lysate. Sup is culture supernatant and cell are cell lysate. (B) THP-1 cells were first treated with LPS for 4 h, and then Hla was added, 4 h later FOM was added. Then the cells and culture supernatant were harvested and western blot were used to detect the expression of NLRP3 inflammasome related-protein. Sup is culture supernatant and cell are cell lysate. (C) The ratio of p-ERK/β-actin, p-P38/β-actin and p-JNK/β-actin and NLRP3 inflammasomes-related protein (ASC, caspase-1 p20, Pro-caspase-1, IL-1β, Pro-IL-1β, IL-18, Pro-IL-18, and NLRP3)/β-actin were calculated. (D) The ratio of NLRP3 inflammasomes-related protein (ASC, caspase-1 p20, Pro-caspase-1, IL-1β, Pro-IL-1β, IL-18, Pro-IL-18, and NLRP3)/β-actin were calculated. Data are means ± standard errors derived from three experiments. * P < 0.05, ** p < 0.01, *** p < 0.0001. [file Image_6.tif]

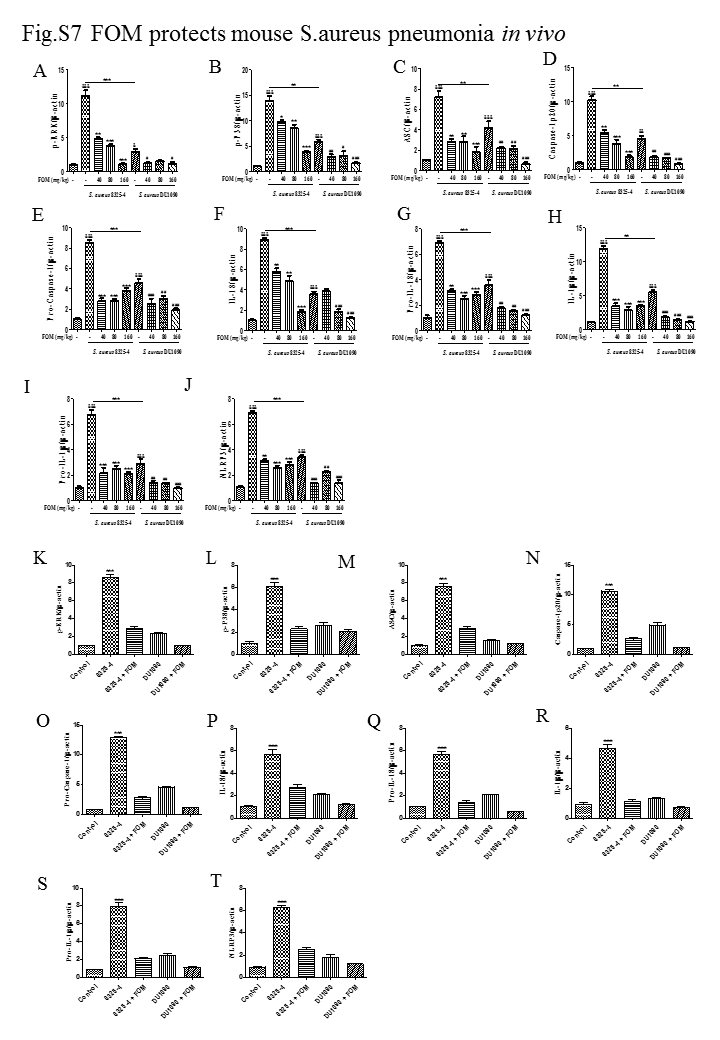

Supplement: Figure S7 — FOM protects mouse S. aureus pneumonia in vivo. (A,K) The ratio of p-ERK/β-actin was calculated. (B,L) The ratio of p-P38/β-actin was calculated. (C,M) The ratio of ASC/β-actin was calculated. (D,N) The ratio of Caspase-1 p20/β-actin was calculated. (E,O) The ratio of Pro-Caspase-1/β-actin was calculated. (F,P) The ratio of IL-18/β-actin was calculated. (G,Q) The ratio of Pro-IL-18/β-actin was calculated. (H,R) The ratio of IL-1β/β-actin was calculated. (I,S) The ratio of Pro-IL-1β/β-actin was calculated. (J,T) The ratio of NLRP3/β-actin was calculated. & p < 0.05, && p < 0.01, &&& p < 0.001 compared with Control group, * p < 0.05, ** p < 0.01, *** p < 0.001 compared with 8325-4 treated group, # p < 0.05, ## p < 0.01, ### p < 0.001 compared with DU1090 treated group for S. aureus pneumonia in vivo. And * P < 0.05, ** P < 0.01, *** P < 0.001 compared with the control groups in the same cell line for SMVs pneumonia in vivo. The data are representative of three experiments with similar results. [file Image_7.tif]
